# Alert for the high prevalence of vitamin D deficiency in adolescents in a large Brazilian sample

**DOI:** 10.1016/j.jped.2024.01.003

**Published:** 2024-03-07

**Authors:** Vanessa Radonsky, Marise Lazaretti-Castro, Maria Izabel Chiamolera, Rosa Paula Mello Biscolla, José Viana Lima Junior, José Gilberto Henriques Vieira, Cynthia Maria Alvares Brandão, Rodrigo Fernandes Ramalho, Sergio Setsuo Maeda, Marcia Wehba Esteves Cavichio

**Affiliations:** a Universidade Federal de São Paulo (UNIFESP), Departamento Endocrinologia, São Paulo, SP, Brazil; b Grupo Fleury, Departamento Endocrinologia, São Paulo, SP, Brazil; c Grupo Fleury, Grupo de Ciências de Dados e Bioinformática, São Paulo, SP, Brazil; d Grupo Fleury, Departamento Pediatria, São Paulo, SP, Brazil

**Keywords:** Vitamin D, Adolescent, 25 Hydroxyvitamin D, Cholecalciferol, Rickets, Childhood

## Abstract

**Objective:**

To estimate the prevalence of vitamin D deficiency and severe deficiency in children and adolescents, in a large Brazilian sample.

**Methodology:**

Results of 413,988 25(OH)D measurements in children and adolescents aged 0 to 18 years collected between 01/2014 and 10/2018 were obtained from the database of a Clinical Laboratory. In this population, 25 hydroxyvitamin D concentrations below 20 ng/mL are considered deficient, and below 12 ng/mL as severe deficiency. All measurements were performed by immunoassay and the results were distributed by gender, age group, seasonality, and latitude.

**Results:**

The mean of 25(OH)D levels was 29.2 ng/mL with a standard deviation of 9.2 ng/mL. Of the total samples, 0.8% had a concentration < 12 ng/mL, and 12.5% of the samples had a concentration < 20 ng/mL, with a higher prevalence in females. Children under 2 years of age had the lowest prevalence. The effects of latitude and seasonality were quite evident. In samples of female adolescents from the southern region in winter, 36% of vitamin D deficiency and 5% of severe deficiency were found.

**Conclusion:**

In this large number of measurements of 25(OH)D in children and adolescents, 12.5% had a deficiency and 0.8% had severe deficiency. A greater deficiency was observed among adolescents, especially females, which raises questions about the need for supplementation during this period of life.

## Introduction

Both in childhood and adolescence, vitamin D plays a fundamental role in the regulation of calcium and phosphorus homeostasis, mainly in mineralization and bone mass acquisition, preventing the appearance of rickets.^1^ Its sources can be through diet or, mainly, by skin synthesis after exposure to ultraviolet light B (UVB - 291 at 315 nm).^2^ After reaching the bloodstream, vitamin D is transported by the vitamin D binding protein (DBP) to the liver where it undergoes a first hydroxylation forming 25 hydroxyvitamin D [25(OH)D] or calcidiol, its main metabolite which is subsequently stored in the liver and body fat and is used for individual assessment of vitamin D status.^3^

Sunlight accounts for about 90 to 95% of the vitamin D supply; whole foods that naturally contain a significant amount of vitamin D are very limited, except for a few oily fish and sun-dried mushrooms, which are not commonly consumed by children and adolescents.^4^ Season, latitude, time of day, skin pigmentation, aging, sunscreen use, and atmospheric pollution can all influence the cutaneous production of vitamin D.^4^

The definition of vitamin D deficiency presents differences between the main groups of specialists. IOM defines vitamin D deficiency as a level of 25 (OH) *D* < 20 ng/mL.^5^ The global consensus on the prevention and management of nutritional rickets considers vitamin D as sufficient when the level of 25(OH)D is greater than 20 ng/mL (50 nmol/L) and deficient with a risk of rickets when it is less than 12 ng/dL (30 nmol/L).^6^

Numerous studies conducted worldwide have demonstrated a high incidence of hypovitaminosis D among children and adolescents.^1^ The prevalence of serum 25(OH)D deficiency in this age group reaches 35% in the United Kingdom and 42% in the USA.^7^^,^^8^ Brazil is a country with vast territorial extension, crossed by the Equator, and 93% of its territory lies in the southern hemisphere. It has a predominance of tropical climates, except for the southern region, which has a subtropical climate. During the summer months, the intensity of ultraviolet radiation is often found at extreme levels across the country on cloudless days and at noon. In the winter, northeastern states such as Pernambuco and Bahia maintain high levels of ultraviolet radiation, but in the southern and southeastern states such as Rio Grande do Sul and São Paulo, a significant reduction is noted. Arantes et al., demonstrated that the concentration of 25 (OH) D in adults in several Brazilian cities was significantly higher in lower latitudes such as in the northeast of the country.^9^ Recently, Borba et al., evaluated 25(OH)D levels in blood samples from healthy adults, collected during the summer in the states of Salvador, São Paulo and Curitiba. They showed a high prevalence of vitamin D deficiency (15.3%) even in the summer months in tropical and subtropical countries like Brazil.^10^

The recent shift in lifestyle habits, particularly after the advent of technology, means that children and adolescents are spending more time engaged in indoor activities such as using computers and smartphones. This trend may induce potential health problems in this population.^11^ Another point to be evaluated is the recognition of the damage caused by the sun's rays on the skin and the use of sunscreens.^12^ Both factors contribute to vitamin D deficiency even in sunny countries like Brazil. However, limited data on the prevalence of hypovitaminosis D are available among Brazilian children and adolescents.

Therefore, the objective of this study was to estimate the prevalence of 25 (OH) D deficiency and severe deficiency in a large Brazilian sample of children and teenagers from north to south of Brazil.

## Materials and methods

Measurements of 25(OH)D of children and adolescents (aging from 0 to 18 years old) were obtained retrospectively from the Fleury Group´s examination database, a supplemental health service, with branches in several Brazilian states, collected between January 2014 and October 2018. Samples with a 25(OH)D above 100 ng/mL and from hospitalized patients were excluded. Samples for 25(OH)D measurements were collected according to the request for routine testing by the assistant physician of the proband. All the measurements of 25(OH)D had been done by chemiluminescence immunoassay (Liason, DiaSorin, Italy), with intra e inter assay coefficient of variation of 6.0% and 8.0%, respectively. External evaluation on DEQAS (the Vitamin D External Quality Assessment Scheme) control sera run for tandem mass spectrum in routine samples and performed blind correlation with the results obtained with the DiaSorin RIA kit. Over the duration analyses, the 25(OH)D assay showed a mean (SD) bias of +5.7% for a total of 30 controls.

The prevalence of 25(OH)D deficiency and severe deficiency were evaluated. Vitamin D deficiency was defined as a 25(OH)D level of less than 20 ng/mL and severe deficiency below 12 ng/mL.

The final dataset was stratified into four categories: i) gender (male and female), ii) Brazilian city of sample collection, iii) the bimester of sample collection, and iv) age ranges. Data were collected from six cities located at different latitudes across the country: Recife (08⁰S), Salvador (12⁰S), Rio de Janeiro (22⁰S), São Paulo (23⁰S), Curitiba (25⁰S), and Porto Alegre (30⁰S). Blood collection times were divided into bimesters: January/February, March/April, May/June, July/August, September/October, and November/December. Four age ranges were defined: 0–24 months, 2–6 years old, 7–10 years old, and 11–18 years old.

The Ethics Committee (CAAE 3543820.1.3001.5474) approved the study, and the informed consent form was waived because the data were anonymized. The authors declare that they have no potential conflicts of interest.

### Statistical analysis

Descriptive statistics are presented as median (25% - 75%), mean (standard deviation) for continuous variables, and frequencies with percentages for categorical variables. The Shapiro and Kolmogorov-Smirnov test was used to assess the normality of quantitative variables showing non-parametric data. The Non-parametric ANOVA (Kruskal Wallis test) was used to compare the means of the dependent. Considered statistically significant *p* < 0.05. To analyze the results, Python3.7 and Rstudio software were used.

## Results

A total of 413,988 dosages of 25 (OH) D were evaluated, and the mean was 29.2 ng/mL with a standard deviation (SD) of 9.2 ng/mL Of the total samples, 12.5% (*n* = 51,748) had vitamin D deficiency, and among those, 0.8% (*n* = 3,312) had severe deficiency. Both conditions were higher in females 14.5% and 0.8% than in males 10.5% and 0.7%, respectively (*p* < 0.05).

The descriptive results and statistical analysis of the mean and median values of 25(OH)D for both sexes according to age ranges, months of the year, and latitudes are shown in Table 1.

**Table 1:** Descriptive statistical distribution of median and mean of 25(OH)D values for categorical variables across age, seasonality and latitude groups, all in both genders.

	**Sex**	**N**	Median (25% - 75%) (ng/mL)	Mean (SD) (ng/mL)	P value
Age					
0 to 24 months	M	12,302	36 (30–43)	37.0 (10.9)	
	F	11,190	35 (29–43)	36.2 (11.3)	
2 to 6 years	M	55,351	31 (26–37)	32.3 (9.0)	
	F	55,064	31 (25–36)	31.5 (8.8)	*P* < 0.05
7 to 10 years	M	47,677	28 (24–34)	29.4 (8.4)	
	F	55,897	27 (23–33)	28.1(8.0)	
11 to 18 years	M	73,267	27 (22–32)	27.6 (8.8)	
	F	103,240	25 (21–31)	26.4 (8.7)	
Bimester					
Jan-Feb	M	34,413	33 (27–40)	33.9 (9.9)	
	F	40,319	31 (26–38)	32.3 (9.5)	
Mar-Apr	M	34,408	31 (26–37)	32.1 (9,4)	
	F	41,759	29 (24–36)	30.6 (9.1)	
May-Jun	M	30,863	28 (23–33)	28.7 (8.5)	
	F	36,547	26 (22–32)	27.3 (8.5)	*P* < 0.05
Jul-Aug	M	36,729	26 (22–32)	27.1 (8.4)	
	F	44,512	25 (20–30)	25.8 (8.3)	
Sep -Oct	M	28,643	28 (23–33)	28.5 (8.7)	
	F	34,335	26 (21–32)	26.9 (8.5)	
Nov-Dec	M	23,541	29 (24–35)	29.9 (8.8)	
	F	27,919	27 (22–33)	28.2 (8.7)	
Latitude					
Recife (08⁰S)	M	11,681	29 (24–35)	31.1 (9.1)	
	F	13,493	27 (22 −33)	29.2 (8.8)	
Salvador (12⁰S)	M	11,062	29 (24–35)	32.3 (9.3)	
	F	12,472	28 (23–33)	30.4 (9.0)	
Rio de Janeiro (22⁰S)	M	31,689	30 (25–36)	30.1 (9.0)	
	F	38,164	28 (23–34)	28.6 (8.7)	*P* < 0.05
São Paulo (23⁰S)	M	103,032	31 (26–38)	29.9 (9.3)	
	F	121,489	29 (24–35)	28.4 (9.1)	
Curitiba (25⁰S)	M	9243	27 (22–33)	28.4 (9.0)	
	F	12,228	26 (21–32)	27.2 (9.1)	
Porto Alegre (30⁰S)	M	21,890	28 (23–35)	29.6 (9.7)	
	F	27,545	27 (22–34)	28.4 (9.5)	

**Figure 1:**
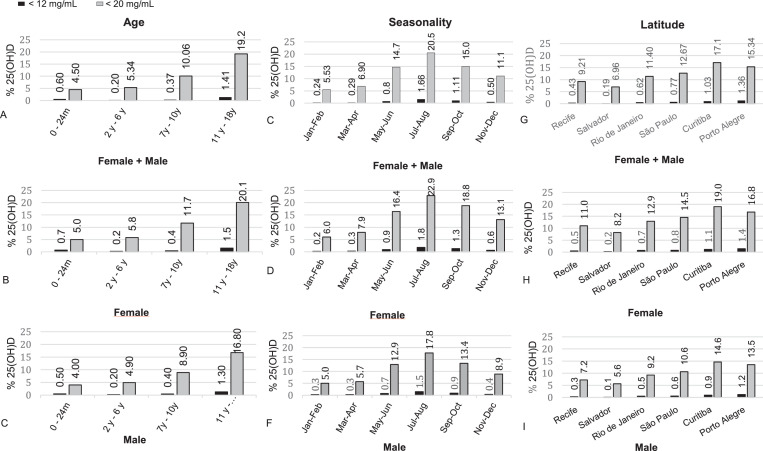
Distribution graph of 25(OH)D deficiency and severe deficiency separated by age, seasonality, latitude and sex.

Children under 2 years had the lowest prevalence of deficiency (4.5%), which increased with age (Figure 1A). In the group of female adolescents aged from 11 to 18 years, 20.1% had vitamin D deficiency, and 1.5% of this group had severe deficiency (Figure 1B). In the male group of the same age, they had 16.8% and 1.3%, respectively (Figure 1C). When this same group of girls was evaluated in the winter months (July/August) in the southern region (between latitudes 25°S and 30°S), 25(OH)D deficiency levels 36% of the samples, 5% with concentration below 12 ng/mL and with a mean of 22.6 ± 7.9 ng/mL. Cities in the northeastern region of the country (Recife and Salvador) had a lower incidence of vitamin D deficiency (9.2 and 6.9%, respectively) when compared to cities in the southern region, such as Curitiba (17.1%) and Porto Alegre (15.3%) (Figure 1F). When the 25(OH)D measurements were analyzed according to seasonality, in the winter months (July/August) deficiency was found in 20.6% and severe deficiency in 1.66% of the total samples, while in the summer months (Jan/Feb) the prevalence was 5.53% and 0.24%, respectively (Figure 1D). All percentage comparisons showed a p-value < 0.05.

## Discussion

This is a descriptive study of the epidemiology of vitamin D deficiency in a large number of 25(OH)D dosages performed in children and adolescents from six different cities in Brazil during the years 2014 to 2018.

There is no consensus on the ideal blood 25(OH)D level in the pediatric population, but most authors agree that a 25(OH)D concentration below 20 ng/mL is indicative of vitamin D deficiency, while a 25(OH)D concentration below 12 ng/mL presents a risk of rickets and osteomalacia.^13^ In this large sample, 12.5% were deficient, and 0.8% of them with severe deficiency. Brazil has a large young population, with more than 53,759,000 individuals with 18 years or less. If the authors transfer our findings to the whole Brazilian young population, there would be about 6720,000 with deficiency and 430.000 with severe deficiency, numbers that should not be negligible.^14^

Similar results have also been observed in other pediatric populations in South America. In Bogota (latitude 4.6°N), Colombia, the prevalence of vitamin D deficiency among girls aged 7 to 11 years was very similar to ours at 11.7%.^15^ In the USA, the prevalence of deficiency in children aged 1 to 11 years was slightly higher at 18%, with severe deficiency at 1%.^16^

WHO data shows a significant discrepancy in the prevalence of vitamin D deficiency across the world. Countries such as Canada, China and Thailand evaluated 25(OH)D levels in children aged 6 to 12 years throughout the year and found a prevalence of deficiency of 24%, 47% and 54%, respectively.^17^ The prevalence found in these countries is significantly higher than ours, which stood at 10% for the same age group (Figure 1A). In addition to geographic and dietary characteristics that strongly influence 25(OH)D concentrations, other aspects, such as skin color, clothing, and lifestyle, must be taken into account to explain the differences in the prevalence of vitamin D deficiency found between different countries.

Data on vitamin D status in the pediatric age group in the Brazilian population are scarce. Melo Bacha et al. also evaluated 25(OH)D levels in 193,725 laboratory samples from children aged 0 to 17 years in cities located between latitudes 14°S and 22°S, and found a prevalence of deficiency levels of 10.5%, ^18^ very similar to ours (11.3%), when analyzing the 25(OH)D levels from cities located between latitudes 12°S and 23°S. Bueno et al., however, showed a lower prevalence of vitamin D deficiency (8.6%) in 58 children and adolescents with short stature aged 4 to 18 years of both sexes living in the city of Porto Alegre (latitude 30°S) throughout the year, lower than the 15.3% the authors found in the same city. This difference could be explained because they excluded children who had some risk factors for vitamin D deficiency, such as chronic diseases and use of some medications.^19^

In the present study, the prevalence of vitamin D deficiency increased with age, being three times more frequent in adolescents than in young children. Among female adolescents, 20.1% of 25(OH)D measurements were within the range of vitamin D deficiency, with 1.5% having severe deficiency. When the group of girls aged 11 to 18 years were evaluated in the months of July/August in the southern region, vitamin D deficiency reached 36% of the samples, of which 5% had a vitamin D concentration below 12 ng/mL. This represents about 820,000 female adolescents with vitamin D deficiency, of whom 140,000 would be severely deficient.^20^ Santos et al. evaluated 25(OH)D levels in girls aged 7 to 18 years throughout the year in cities in southern Brazil and found an even higher prevalence of vitamin D deficiency, 36.3%. In the present study, the year-long prevalence of vitamin D deficiency for this age group was 22,5%. This difference could be explained by the samplings. In Santos et al., the girls were from public schools with lower income, and none of them was taking supplementation. This may suggest that vitamin D deficiency may be more evident in vulnerable groups.^21^

Other studies have also reported an increase in the prevalence of vitamin D deficiency during adolescence compared to younger children. In Rio de Janeiro, Brazil, Leao et al. also observed a significant decrease in 25(OH)D levels in the age group of 12 to 18 years when compared to the age group of 1 to 11 years.^22^ Similarly, this phenomenon was observed in children and adolescents in a meta-analysis conducted on several European cohorts, which included individuals aged 1 to 18 years. They reported a prevalence of vitamin D deficiency ranging from 4 to 7% in the group of children aged 1 to 6 years old, and reaching 12 to 40% in the group of adolescents aged 15 to 18 years old.^23^

These findings are concerning because adolescence is a critical period for bone mineral accumulation, with bone formation predominating over bone resorption to allow for bone growth. Many factors will influence bone mass gain during growth, including genetic factors, sex, endocrine factors, nutritional factors (calcium, protein, and vitamin D), mechanical forces, and exposure to risk factors such as alcohol and smoking.^24^ To achieve a good peak of bone mass (PBM) at the end of skeletal maturation, it is essential to maintain sufficient levels of vitamin D, which directly regulates calcium and phosphorus homeostasis, critical elements for normal bone mineralization.^24^ Puberty plays a key role in the acquisition of PBM, as skeletal mass doubles between the onset of puberty and adulthood. PBM is a lifelong determinant of osteoporosis and fragility fractures.^25^ Pan et al., demonstrated that in adolescents aged 12 to 19 years, 25(OH)D levels have a positive correlation with total BMD.^26^ Therefore maintaining normal levels of vitamin D and calcium intake during this period of life seems to be an appropriate measure to reduce the risk of osteoporosis and fractures in the future.

There are several causes for the increase in vitamin D deficiency in adolescents, including school and work demands leading to reduced opportunities for sun exposure, as well as the group's characteristic behavior of staying indoors more often. Additionally, the authors can highlight the increased prevalence of obesity in this age group, along with the use of sunscreen and the lower intake of vitamin D or supplements.

Vitamin D supplementation up to 2 years of age is a well-established practice among Brazilian pediatricians. However, there is no consensus on supplementation beyond this age. These results confirm this practice, showing a much lower prevalence of vitamin D deficiency in children under 2 years of age (4.5% deficiency and 0.6% severe deficiency) compared to adolescents (18.4% deficiency and 1,4% severe deficiency). The Brazilian Society of Pediatrics recommends vitamin D supplementation with 400 IU/day for children up to 1 year of age and 600 IU/day for children and adolescents over 1 year of age who do not consume at least 600 IU of vitamin D per day in their diet, have no regular sun exposure, or have risk factor to vitamin D deficiency.^27^ Giustina et al., at the Third International Vitamin D Conference in 2020, recommended supplementation of 400 to 600 IU of vitamin D per day in the pediatric age group with the aim of preventing rickets.^28^ Some European societies have recommended vitamin D in children and adolescents during the winter months or throughout the year if exposure to the sun is reduced in other months.^29^^,^^30^

Some limitations of this study must be recognized. The samples belong to a private health network population, and the authors do not have information related to the use of vitamin D supplementation, use of sunscreen, ethnicity, obesity, or underlying diseases. So, generalization for the entire Brazilian population in this age group must be done with caution. Despite this, some strengths should be highlighted, such as the large number of 25(OH)D measurements, all measured with the same methodology, in different age groups, sex, latitudes and times of the year. Thus, the results show a good view of the prevalence of vitamin D deficiency in the children and adolescents.

Guiding more and safe sun exposure and/or vitamin D supplementation for the studied pediatric and adolescent populations is essential for skeletal growth and development. But how far can we encourage increased sun exposure? The application of sunscreens in childhood is a strong recommendation from several scientific organizations aimed at preventing sunburn and skin cancer.^12^ If exposure to sunlight directly on the skin should be avoided in any situation, as advocated by dermatology societies, based on the present findings the authors encourage vitamin D supplementation throughout longitudinal growth, especially for female adolescents, even in a sunny country like Brazil.

## Conclusion

The present study was the first to describe the concentration of 25(OH)D in a representative number of samples from children and adolescents in six different cities in a sunny country like Brazil. Even without information on the use of supplementation or sunscreen, the presence of vitamin D deficiency occurred in 11.7% of the samples, with 0.8% of severe deficiency being more prevalent in the winter months, in cities with greater latitude, and in older age. In the group of female adolescents in the months of July/August in the southern region, 36% of 25(OH)D dosages were below 20 ng/mL and 5% below 12 ng/mL. Pediatricians should be aware of the importance of vitamin D in the health of children especially female adolescents. Encouraging vitamin D supplementation in this group with regular recommended doses (400 to 600 IU/daily) must be essential to prevent osteoporosis and its consequences in the future of this youth.

## Conflicts of interest

The authors declare no conflicts of interest.

## References

[1] Antonucci R., Locci C., Clemente M.G., Chicconi E., Antonucci L. (2018). Vitamin D deficiency in childhood: old lessons and current challenges. J Pediatr Endocrinol Metab.

[2] Holick M.F. (2013). Caballero b. Encyclopedia of Human Nutrition.

[3] Hossein-nezhad A., Holick M.F. (2013). Vitamin D for health: a global perspective. Mayo Clin Proc.

[4] Bouillon R., Marcocci C., Carmeliet G., Bikle D., White J.H., Dawson-Hughes B. (2019). Skeletal and extraskeletal actions of vitamin D: current evidence and outstanding questions. Endocr Rev.

[5] Rosen C.J., Abrams S.A., Aloia J.F., Brannon P.M., Clinton S.K., Durazo-Arvizu R.A. (2012). IOM committee members respond to Endocrine Society vitamin D guideline. J Clin Endocrinol Metab.

[6] Munns C.F., Shaw N., Kiely M., Specker B.L., Thacher T.D., Ozono K. (2016). Global Consensus Recommendations on Prevention and Management of Nutritional Rickets. J Clin Endocrinol Metab.

[7] Absoud M., Cummins C., Lim M.J., Wassmer E., Shaw N. (2011). Prevalence and predictors of vitamin D insufficiency in children: a Great Britain population based study. PLoS One.

[8] Gordon C.M., DePeter K.C., Feldman H.A., Grace E., Emans S.J (2004). Prevalence of vitamin D deficiency among healthy adolescents. Arch Pediatr Adolesc Med.

[9] Arantes H.P., Kulak C.A., Fernandes C.E., Zerbini C., Bandeira F., Barbosa I.C. (2013). Correlation between 25-hydroxyvitamin D levels and latitude in Brazilian postmenopausal women: from the Arzoxifene Generations Trial. Osteoporos Int.

[10] Borba V.Z., Lazaretti-Castro M., Moreira S.D., de Almeida M.C., Moreira E.D. (2022). Epidemiology of vitamin D (EpiVida)-a study of vitamin D status among healthy adults in Brazil. J Endocr Soc.

[11] Dresp-Langley B. (2020). Children's health in the digital age. Int J Environ Res Public Health.

[12] Balk S.J., Council on Environmental Health; and Section on Dermatology (2013). Ultraviolet radiation: a hazard to children and adolescents (technical report). Pediatr Clin Pract Guidelines Policies.

[13] Holick M.F., Chen T.C. (2008). Vitamin D deficiency: a worldwide problem with health consequences. Am J Clin Nutr.

[14] IBGE Censo 2010. [cited 13 Sep 2022]. Available: https://censo2010.ibge.gov.br/sinopse/index.php?dados=12

[15] Villamor E., Marin C., Mora-Plazas M., Baylin A. (2011). Vitamin D deficiency and age at menarche: a prospective study. Am J Clin Nutr.

[16] Mansbach J.M., Ginde A.A., Camargo C.A. (2009). Serum 25-hydroxyvitamin D levels among US children aged 1 to 11 years: do children need more vitamin D?. Pediatrics.

[17] World Health Organization. A review of disease burden, causes, diagnosis, prevention and treatment. [cited 31 Dec 2023]. Available from:https://apps.who.int/iris/bitstream/handle/10665/329859/9789241516587-eng.pdf

[18] de Melo Bacha F.V., Gomez F.L., Silva A.L., Reis M.D., Cabral E.D., de Carvalho L.D. (2022). Vitamin D: a 14-year retrospective study at a clinical laboratory in Brazil. Arch Endocrinol Metab.

[19] Bueno A.L., Czepielewski M.A., Raimundo F.V. (2010). Calcium and vitamin D intake and biochemical tests in short-stature children and adolescents. Eur J Clin Nutr.

[20] IBGE Censo 2010. [cited 13 Sep 2022]. Available: https://censo2010.ibge.gov.br/sinopse/index.php?dados=12

[21] Santos B.R., Mascarenhas L.P., Satler F., Boguszewski M.C., Spritzer P.M. (2012). Vitamin D deficiency in girls from South Brazil: a cross-sectional study on prevalence and association with vitamin D receptor gene variants. BMC Pediatr.

[22] Leão L.M., Rodrigues B.C., Dias P.T., Gehrke B., Souza T.D., Hirose C.K. (2021). Vitamin D status and prevalence of hypovitaminosis D in different genders throughout life stages: a Brazilian cross-sectional study. Clinics.

[23] Cashman K.D., Dowling K.G., Škrabáková Z., Gonzalez-Gross M., Valtueña J., De Henauw S. (2016). Vitamin D deficiency in Europe: pandemic?. Am J Clin Nutr.

[24] Stagi S., Cavalli L., Iurato C., Seminara S., Brandi M.L., de Martino M. (2013). Bone metabolism in children and adolescents: main characteristics of the determinants of peak bone mass. Clin Cases Miner Bone Metab.

[25] Maggioli C., Stagi S. (2017). Bone modeling, remodeling, and skeletal health in children and adolescents: mineral accrual, assessment and treatment. Ann Pediatr Endocrinol Metab.

[26] Pan K., Tu R., Yao X., Zhu Z. (2021). Associations between serum calcium, 25(OH)D level and bone mineral density in adolescents. Adv Rheumatol.

[27] Sociedade Brasileira Pediatria. Departamento Científico de Endocrinologia. Guia prático de atualização. Hipovitaminose D em pediatria: recomendações para o diagnóstico, tratamento e prevenção. [cited 20 Feb 2024]. Available from:https://www.sbp.com.br/fileadmin/user_upload/2016/12/Endcrino-Hipovitaminose-D.pdf.

[28] Giustina A., Bouillon R., Binkley N., Sempos C., Adler R.A., Bollerslev J. (2020). Controversies in vitamin D: a statement from the third international conference. JBMR Plus.

[29] Płudowski P., Karczmarewicz E., Bayer M., Carter G., Chlebna-Sokół D., Czech-Kowalska J. (2013). Practical guidelines for the supplementation of vitamin D and the treatment of deficits in Central Europe - recommended vitamin D intakes in the general population and groups at risk of vitamin D deficiency. Endokrynol Pol.

[30] Vidailhet M., Mallet E., Bocquet A., Bresson J.L., Briend A., Chouraqui J.P. (2012). Vitamin D: still a topical matter in children and adolescents. A position paper by the Committee on Nutrition of the French Society of Paediatrics. Arch Pediatr.

